# Differential Glycosylation and Modulation of Camel and Human HSP Isoforms in Response to Thermal and Hypoxic Stresses

**DOI:** 10.3390/ijms19020402

**Published:** 2018-01-30

**Authors:** Abdullah Hoter, Mahdi Amiri, Abdelbary Prince, Hassan Amer, Mohamad Warda, Hassan Y. Naim

**Affiliations:** 1 Department of Physiological Chemistry, University of Veterinary Medicine Hannover, 30559 Hannover, Germany; abdullah.hoter@tiho-hannover.de or abdo_hotar@yahoo.com (A.H.); amiri.mahdi@mh-hannover.de (M.A.); 2 Department of Biochemistry and Chemistry of Nutrition, Faculty of Veterinary Medicine, Cairo University, Giza 12211, Egypt; bioproteomics@yahoo.com (A.P.); hassanabd47@hotmail.com (H.A.); maawarda@hotmail.com (M.W.)

**Keywords:** camel, heat shock proteins, HSPA6, CRYAB, *O*-GlcNAc, hypoxia, heat stress, protein expression

## Abstract

Increased expression of heat shock proteins (HSPs) following heat stress or other stress conditions is a common physiological response in almost all living organisms. Modification of cytosolic proteins including HSPs by *O*-GlcNAc has been shown to enhance their capabilities for counteracting lethal levels of cellular stress. Since HSPs are key players in stress resistance and protein homeostasis, we aimed to analyze their forms at the cellular and molecular level using camel and human HSPs as models for efficient and moderate thermotolerant mammals, respectively. In this study, we cloned the cDNA encoding two inducible HSP members, HSPA6 and CRYAB from both camel (*Camelus dromedarius*) and human in a Myc-tagged mammalian expression vector. Expression of these chaperones in COS-1 cells revealed protein bands of approximately 25-kDa for both camel and human CRYAB and 70-kDa for camel HSPA6 and its human homologue. While localization and trafficking of the camel and human HSPs revealed similar cytosolic localization, we could demonstrate altered glycan structure between camel and human HSPA6. Interestingly, the glycoform of camel HSPA6 was rapidly formed and stabilized under normal and stress culture conditions whereas human HSPA6 reacted differently under similar thermal and hypoxic stress conditions. Our data suggest that efficient glycosylation of camel HSPA6 is among the mechanisms that provide camelids with a superior capability for alleviating stressful environmental circumstances.

## 1. Introduction

Arabian camels (*Camelus dromedarius*) live in extremely harsh conditions with periods of extended water deprivation, high ambient temperatures and exposure to direct sunlight in summer days, as well as very cold temperatures during winter nights, all requiring remarkable physiological characteristics and adaptive responses to allow survival [1,2]. *Camelus dromedarius* is a homothermic animal which is able to fluctuate its body temperature up to 42 °C in response to extensive heat stress [3,4]. Previous functional studies showed that camel fibroblasts are more resistant to high temperatures compared to the same cell type from mouse origin [4]. In addition, the expression of HSP73 in camel lymphocytes was significantly increased after heat shock [5]. Therefore, camels are interesting species for studying specific roles of HSP proteins relevant to normal cellular physiology.

Heat shock proteins (HSPs) are stress responsive molecules that control numerous crucial pathways inside the eukaryotic cells. Folding of proteins, hormone signaling, cell cycle control and apoptosis are eminent examples of processes orchestrated by HSPs [6,7,8]. Besides their putative chaperone roles in protein folding, HSPs prevent the accumulation of mis- or unfolded proteins in the cell [9,10,11]. Upon exposure to high temperature or other stress conditions like glucose deficit, hypoxia and infection, or pathological conditions including cancer, expression of these proteins is increased inside the cells and the ability of different organisms to tolerate the unfavorable conditions is significantly enhanced [12,13]. 

αB-crystallin (CRYAB or HSPB5) is a stress inducible chaperone which was initially recognized as lens protein and later proved as an essential chaperone in different tissues [14,15]. Heat stress is an important factor in CRYAB overexpression specially in cardiac tissue [16,17]. CRYAB binds to partially unfolded proteins in an ATP-independent manner to preserve them in a soluble status and so avoid intracellular protein aggregation [18]. The functional multiplicity of CRYAB in different tissues is mainly attributed to its posttranslational modification [19]. This protein is highly expressed in hypoxic areas of the tumor [20], plausibly enhancing the survival capability of hypoxic cells. Interestingly, small HSPs, such as CRYAB, are reported to exhibit differential cellular localization patterns upon thermal stress in cardiac myocytes [21].

Members of the HSP70 family are well recognized to protect prokaryotic as well as mammalian cells from thermal stress or hypoxic stresses [12,22,23]. HSPA6 is a HSP70 chaperone that is induced after severe cellular stress [24]. Induction of HSPA6 has been employed as a tool for detection of cytotoxicity [25,26]. Although the gene encoding HSPA6 is present in humans, it is absent in rodents [27]. In fact, HSPA6 is strictly regulated and highly homologous to HSP70 [24]. However, distinct functions between the two aforementioned chaperones were identified [28,29]. In addition, expression on the surface of certain colon cell lines in response to proteasome inhibition [30] and localization to the sites of transcription in human neurons following thermal stress [31] are interesting characteristics of the HSPA6 protein. Additionally, overexpression of HSPA6 has been linked to progress and development of diseases including atherosclerosis and cancer [32,33].

The current study focused on the analysis of the camel αB-crystallin (CRYAB) and HSPA6 as well as the comparison of their structural and posttranslational processing to their peers in human in response to thermal and hypoxic stress conditions. Since HSPs, including members of HSP70 family, were tested using COS-1 cells in previous studies [34,35], we used these cells as an independent cell model for investigating the processing and posttranslational modifications of camel and human HSPA6 as well as CRYAB orthologues under similar conditions. While the recombinant forms of camel and human CRYAB did not show marked differences by Western blot analysis, interestingly, *O*-linked β-*N*-acetylglucosamine (*O*-GlcNAc) glycosylation, which has been shown to be directly linked to the cellular capability to withstand lethal stresses [36,37], revealed different expression isoforms and variant glycosylation patterns in camel HSPA6 as compared to the human homologue. The current study provides an evidence for differential structural and posttranslational processing between camel and human HSPA6, besides variant response to heat as well as hypoxic stress of the two species. The structural characteristics and posttranslational modifications in CRYAB and HSPA6 are suggested to play a role in the efficient proteostasis and thermotolerance in camelids.

## 2. Results

### 2.1. Cloning, Expression and Localization of Camel and Human HSPs in COS-1 Cells

**Figure 1:**
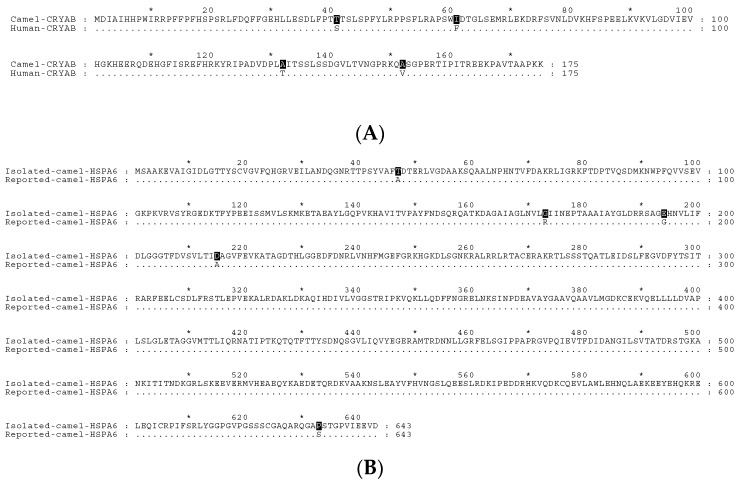

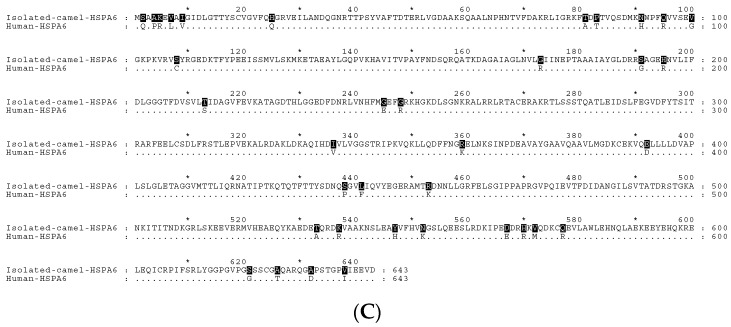
Homology analysis of the deduced amino acid sequences of cDNA clones of camel and human HSPs. Sequence alignment was produced by Clustal Omega and edited using GeneDoc version 2.7.000. (**A**) Amino acid alignment of camel and human CRYAB showing the four different amino acids shaded in black; (**B**) Alignment of isolated camel HSPA6 (GenBank, MG021195) with reported camel HSPA6 sequence (GenBank, ADO12067.1) showing amino acid variations at five amino acids as indicated by the black shaded positions; (**C**) Amino acid alignment of isolated camel HSPA6 (GenBank, MG021195) and human HSPA6 showing variant amino acids shaded in black. An asterisk indicates a number of ten amino acid residues while dots in the lower lines designate identical amino acid residues to the corresponding upper ones.

**Figure A1:**
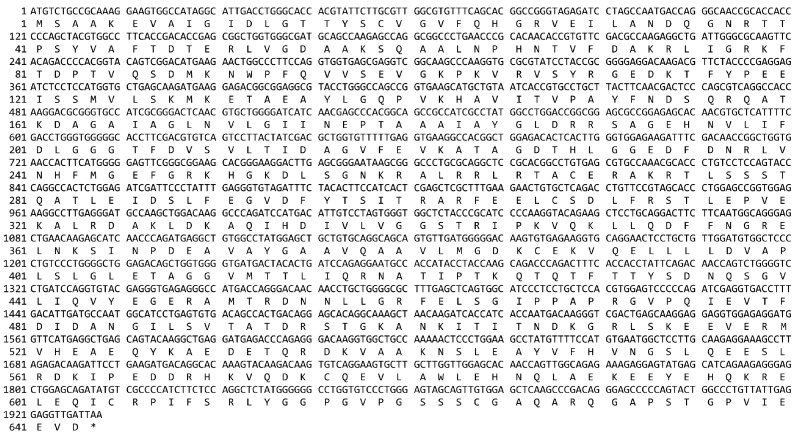
The cDNA sequence of camel HSPA6 (accession number, MG021195). The upper lines represent the cDNA sequence while the lower lines refer to the deduced amino acids.

The cDNAs encoding camel and human CRYAB and HSPA6 were isolated from liver tissue of the *Camelus dromedarius* and human Caco-2 cells by PCR. The PCR products revealed the characteristic length for each camel or human HSP candidate. For instance, both camel CRYAB (cCRYAB) and human CRRYAB (hCRYAB) revealed 528 bp products while cHSPA6 and hHSPA6 showed PCR product lengths of 1932 bp. The deduced primary protein sequence of camel and human αB-crystallin exhibited strong similarities of 98.3% at the amino acid level with only four amino acids being different between the two species. The different amino acids are p.Thr41Ser, p.Ile61Phe, p.Ala132Thr and p.Ala152Val (Figure 1A). Interestingly, we have isolated a cDNA clone of cHSPA6 that differs from the reported camel HSPA6 clone (ADO12067.1) in the NCBI database. The obtained cDNA of camel HSPA6 was sequenced, submitted to the GenBank database and gained the accession number (MG021195). Figure A1 demonstrates the sequence of the amplified camel HSPA6 cDNA clone with its deduced amino acids. The deduced protein of the camel HSPA6 cDNA clone (accession number, MG021195) revealed five amino acid substitutions in comparison to the reported *Camelus dromedarius* HSPA6 (ADO12067.1) with identity and similarity scores of 99.2%. The variant amino acids are p.Ala47Thr, p.Arg173Gly, p.Gly194Glu, p.Ala215Asp, p.Ser633Pro (Figure 1B). On the other hand, the isolated cHSPA6 reflected 94.4% identity and 96.9% similarity with hHSPA6 where there were few differences in the amino acid sequence (Figure 1C). In addition, the isolated *Camelus dromedarius* cHSPA6 revealed 99.7% similarity and 99.4% identity with the two humped camel species (*Camelus ferus*) (NCBI Reference Sequence (XM_006173149.2)).

**Figure 2:**
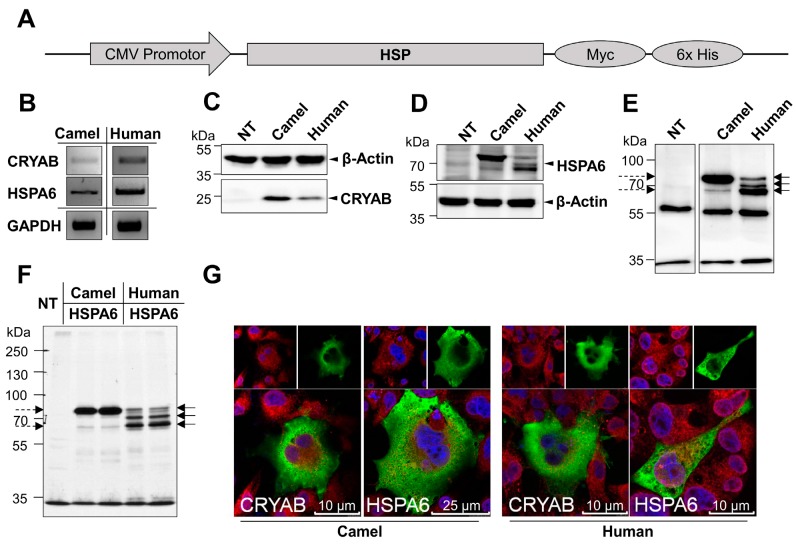
Cloning, expression and localization of *Camelus dromedarius* and human HSPs: (**A**) Schematic diagram showing the general design of cloned camel and human HSPs. Camel and human CRYAB or HSPA6 are Myc-tagged in their C-terminal and their expression is controlled by cytomegalovirus (CMV) promotor; (**B**) The upper and middle panels show *Camelus dromedarius* and human CRYAB as well as HSPA6 amplified cDNA products with sizes of 1932 bp and 528 bp, respectively. Amplified GAPDH cDNA product in the lower panel was used as control for RNA isolation and cDNA synthesis; (**C**,**D**) Western blot analysis of proteins analyzed by SDS-PAGE on 9% slab gels showing expression of camel and human CRYAB and HSPA6 respectively after transfection in COS-1 cells. Camel and human CRYAB were detected as protein bands of an apparent molecular weight of 25 kDa while camel and human HSPA6 specific bands run at around 70 kDa on the gel. Non-transfected COS-1 cells (NT). Actin, a house keeping protein was used as a loading control; (**E**) Immunoprecipitation of camel and human HSPA6 showing different isoforms between the two HSPs upon expression in COS-1 cells. As illustrated, camel HSPA6 (dashed arrows) has two isoforms, a highly expressed high molecular weight form and a lower molecular weight faint one while the human HSPA6 (continuous arrows) reveals three protein bands with the lowest being the most expressed isoform; (**F**) Immunoprecipitation of ^35^S labeled Myc tagged camel and human HSPA6 confirms the results in C; (**G**) Laser confocal microscopy reveals camel and human HSPA6 and CRYAB in the cytosol of transfected COS-1 cells. HSP (green) was visualized together with the cell nucleus (blue) and calnexin (red) as an ER marker. Scale bars: 25 μm or 10 μm.

**Figure A2:**
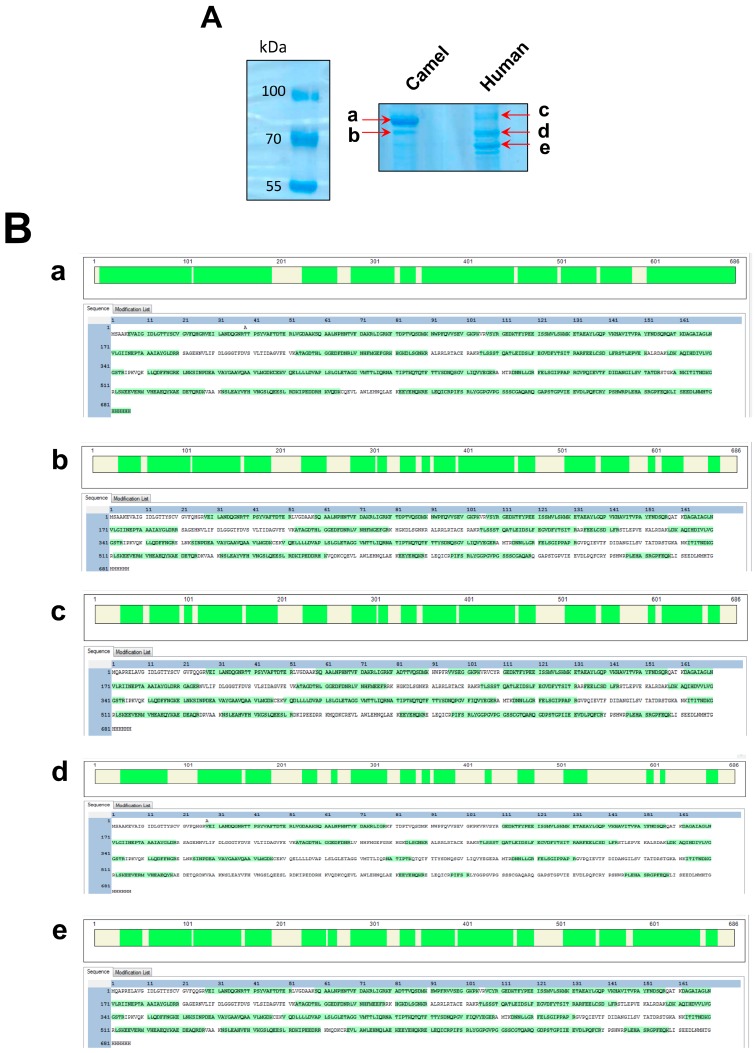
Expression and mass spectrometry analysis of camel and human HSPA6. (**A**) 9% SDS showing the expressed protein forms of recombinant camel HSPA6 (cHSPA6) and human HSPA6 (hHSPA6) after transfection of COS-1 cells and IP using anti-Myc antibody. The cHSPA6 revealed 2 isoforms (**a**,**b**) while hHSPA6 showed 3 isoforms (**c**–**e**); (**B**) Mass Spectrometry results of the excised cHSPA6 and hHSPA6 protein bands. The green coloration indicates the coverage of the analyzed peptides.

The cDNAs of camel and human HSPs obtained by PCR (Figure 2B) were successfully cloned into the Myc-tagged mammalian expression vector (pcDNA3.1 Myc His A) and their identities were assessed by restriction digestion and sequencing. Camel and human cDNA constructs were transfected into COS-1 cells. The expressed proteins were thereafter either immunoprecipitated or directly analyzed by Western blots using anti-Myc antibody. The Myc-tagged cCRYAB and hCRYAB were identified as single protein bands of about 25 kDa (Figure 2C). The proteins of cHSPA6 and hHSPA6 revealed different isoforms slightly higher or at the same level of 70 kDa (Figure 2D,E). Variations between the camel and human HSPA6 were detected. The camel HSPA6 showed two bands of variable intensities with high density and low faint bands, while the human HSPA6 comprised thee isoforms including a lower density band. Similar results were obtained in biosynthetic labeling experiments (Figure 2F). Finally, mass spectrometry analysis was carried out after excision and purification of the corresponding camel and human HSPA6 bands (Figure A2). The cHSPA6 and hHSPA6 peptides showed 65–86% coverage of camel HSPA6 and 67–74% coverage of the human HSPA6 as listed in Table A1.

We then examined the intracellular localization of the four recombinant HSPs in transfected COS-1 cells. All analyzed HSPs exhibited similar cellular localization predominantly in the cytosol and to a certain extent also in the nucleus (Figure 2G).

### 2.2. Glycosylation of Camel and Human HSPs

Using Yin-o-Yang server (Available on: http://www.cbs.dtu.dk/services/YinOYang/) which estimates *O*-β-GlcNAc binding sites in eukaryotic protein sequences, camel and human CRYAB showed nearly similar distribution of predicted *O*-GlcNAc sites with eight and nine Ser/Thr sites, respectively. Unlike the *O*-GlcNAc similarity in cCRYAB and hCRYAB, camel HSPA6 was predicted to contain nine Ser/Thr sites compared to five Thr sites in human HSPA6 as shown in Table 1.

**Figure 3:**
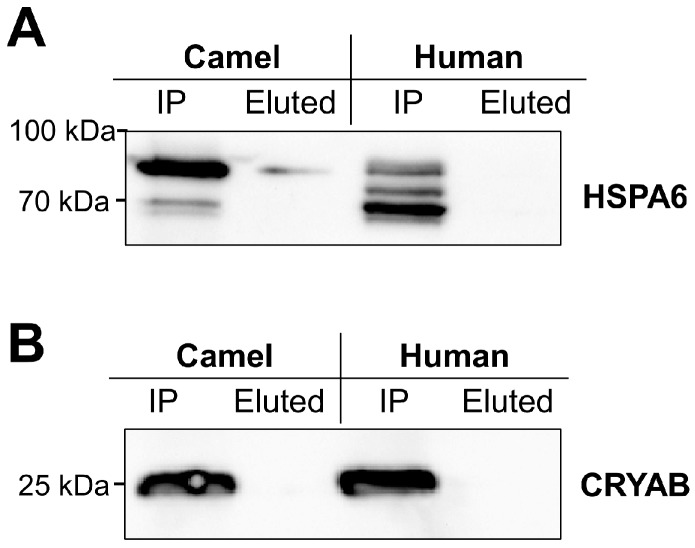
Differential glycosylation of camel and human HSPA6. (**A**) *Helix pomatia* (lectin)-conjugated Sepharose was used to discriminate between the glycan structures of the Myc-tagged camel and human HSPA6 proteins. The extracted proteins from camel/human HSPA6-transfected COS-1 cells were mixed with *Helix pomatia* lectin-Sepharose for 2–3 h and the bound proteins were eluted with 10 mM *N*-acetyl-glucosamine. Camel HSPA6, but not the human species bound to the lectin. As a positive control, immunoprecipitation (IP) of camel and human HSPA6 was performed; (**B**) Similar experiment was performed for the Myc-tagged camel and human CRYAB. In contrast to camel HSPA6, both chaperones did not bind to the lectin. HSPA6 and CRYAB were detected by anti-Myc antibody. The Western blot shown is representative of three experimental independent repeats.

Based on these predictions, we wanted to elucidate the *O*-glycosylation patterns of camel and human HSPA6 by assessment of their binding capacities to the *Helix pomatia* lectin, which is highly specific towards *O*-linked *N*-acetyl-galactosamine, but binds also fairly well *N*-acteyl-glucosamine [38]. As shown in the Western blot, detectable amounts of camel HSPA6 bound to *Helix pomatia* lectin and were eluted with the specific sugar *N*-acetyl-glucosamine (Figure 3A, left panel). By contrast, human HSPA6 did not bind to the lectin under normal or stress conditions (longer exposure of the blot did not reveal a protein band in the eluted sample of human HSPA6, see Appendix C). We have also examined the binding of the camel CRYAB and the human CRYAB to *Helix pomatia* lectin. As shown in Figure 3B, neither chaperone was detected in the sugar protein eluates indicating that these two chaperones are not *O*-glycosylated in this experimental set-up.

### 2.3. Response of Camel and Human HSPA6 Isoforms to Heat and Low Oxygen Stress Conditions 

**Figure 4:**
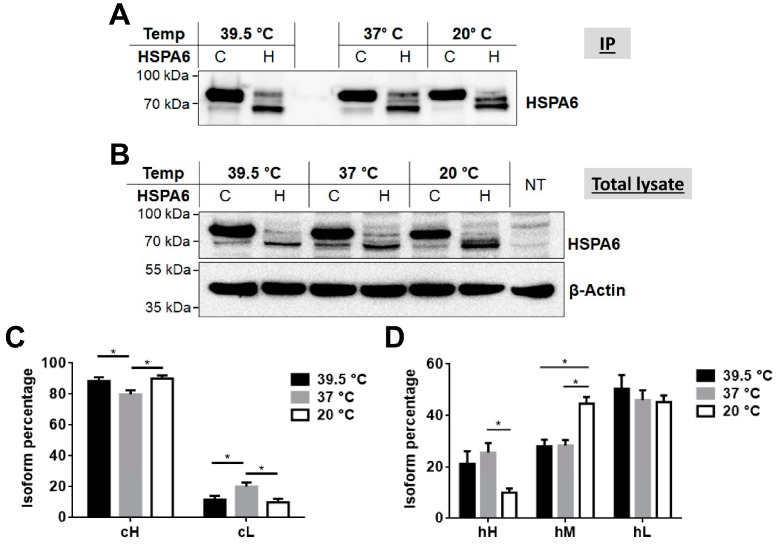
Variable response of camel and human HSPA6 isoforms to different temperatures. (**A**) Western blots of immunoprecipitated camel (C) or human (H) HSPA6 proteins from transfected COS-1 cells and exposure to different temperatures (39.5 °C, 37 °C and 20 °C); (**B**) The loading controls for (A) utilized equal amount of proteins in lysates of transfected COS-1 cells; (**C**,**D**) Quantification of each camel/human HSPA6 isoform in the total protein isoforms. Camel HSPA6 is identified by two isoforms (higher (cH) and lower (cL)) while human HSPA6 exhibits three isoforms (higher (hH), middle (hM) and lower (hL)). Statistically significant values are denoted by an asterisk and the blots are representative data sets out of five independent repeats.

**Figure 5:**
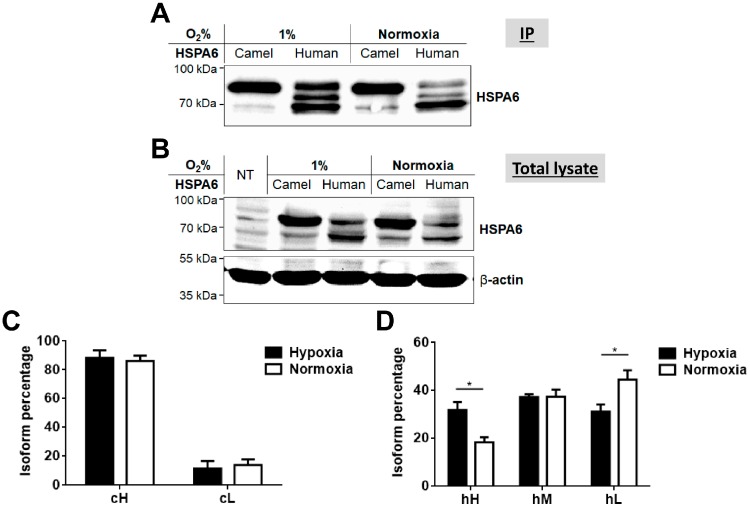
Response of camel and human HSPA6 isoforms to different oxygen concentrations. (**A**) 24 h after transfection, cells were cultured under normal or 1% oxygen levels for 15 h followed by immunoprecipitation of HSPA6 and Western blotting; (**B**) The loading controls for (A) utilized equal amount of proteins in lysates of transfected COS-1 cells; (**C**,**D**) Quantification of each camel or human HSPA6 isoform in the total protein isoforms after incubation under hypoxic or normoxic conditions. As described in Figure 4, camel HSPA6 is identified by two isoforms (higher (cH) and lower (cL)), while human HSPA6 exhibits three isoforms (higher (hH), middle (hM) and lower (hL)). Statistically significant values are denoted by an asterisk and the blots are representative data sets out of five independent repeats.

In view of the variations in the molecular forms of the camel and human HSPA6 proteins we asked whether the HSPA6 isoforms are regulated at the posttranslational level in response to stress conditions. For this purpose, the expression of these proteins was examined by subjecting the cells to different temperatures and also to hypoxic conditions. Although both camel and human HSPA6 isoforms responded to both thermal and low oxygen stress conditions, the higher molecular weight isoform of cHSPA6 (cH) was dominant under all applied stress conditions as compared to the hHSPA6 (Figure 4 and Figure 5). Quantification of the protein bands expressed under these conditions showed that the level of the lower molecular weight form of camel HSPA6 (cL) decreased at 20 °C and 39.5 °C as compared to 37 °C, whereas that of the higher main isoform (cH) increased under the same conditions (Figure 4A,C). Human HSPA6 followed another pattern since the level of the lower molecular weight isoform (hL) increased at 39.5 °C and the middle isoform (hM) increased markedly at 20 °C with a concomitant reduction in the level of the higher isoform (hH) (Figure 4A,D). When COS-1 cells expressing the camel and human HSPA6 were subjected to variable oxygen levels, comparable changes in the expression level of the isoforms were observed (Figure 5). These changes included slightly decreased levels of the cL form after 1% oxygen incubation and marked increased expression level of hH.

## 3. Discussion

HSPs are a cluster of highly homologous proteins that promote the tolerance to environmental and cellular stresses in mammals. It is well known that camels are naturally equipped with physiological particularities to cope with the harsh environmental conditions. Gross anatomical features of camels revealed adaptive mechanisms by which camels tolerate elevated temperatures. For instance, during heat stress selective brain cooling, which involves the organization of superficial veins, the conformation of the nasal cavity and the arterial blood supply all contribute to preserving brain temperature below central blood temperature [2]. Camels, like other desert animals are subject to proteotoxic stresses such as exposure to prolonged high temperatures during summer and very low temperature during winter, besides other stresses which require molecular adaptation to such extreme circumstances [1,3]. The molecular mechanisms contributing to cellular stress adaptation including post translational modification of HSPs are not fully explored. Comparative molecular and cellular analyses of the camel HSPs with their human counterparts or other species can expand and contribute to our knowledge on the potential mechanisms that govern and affect the function of HSPs in different species.

In this study, we analyzed the expression pattern and *O*-GlcNAc modifications of two inducible HSPs belonging to HSP20 and HSP70 families from both camel and human species under normal and stress conditions including heat and hypoxic stress. These HSPs, CRYAB and HSPA6, are reported to have increased expression and altered localization following thermal or hypoxic stress [17,39,40,41]. Our data show that the natural variant of camel HSPA6 clone is quite similar to the reported sequence (ADO12067.1) on the NCBI database. Pairwise alignment of our translated cDNA isolate exhibited 99.4% identity and 99.7% similarity with the two humped camel species *(Camelus ferus)*, NCBI (XP_006173211.1) suggesting high functional and structural relationships between camel species. Interestingly, despite the extreme similarities (amino acid identity 94.4%) between camel and human HSPA6, variations in their protein expression patterns could be discerned due to differences in their posttranslational modifications. In fact, database analyses and subsequent lectin pull-down assays revealed increased cytosolic *O*-glycosylation of camel HSPA6 as compared to its human counterpart. In the experimental set-up utilized, the hHSPA6 isoforms showed no evidence for *O*-glycosylation with GlcNAc while the camel orthologue did. Nevertheless, the level of *O*-GlcNAc glycosylated cH isoform of cHSPA6 eluted from the *Helix pomatia* lectin was low as compared to the high expression level of this form. This is presumably due to potential unfavorable locations of the *O*-linked glycosylation sites within folded structures of the chaperone resulting in a limited exposure of a short monosaccharide to the lectin. In addition, *Helix pomatia* lectin exhibits a preferential binding specificity towards *O*-linked GalNAc and to a lesser extent *O*-GlcNAc [38]

Along similar lines, the lack of binding of potential *O*-GlcNAc sites in hHSPA6 to *Helix pomatia* lectin can be explained. Moreover, in silico predictions of potential *O*-linked glycosylation sites do not a priori warrant that these sites could be posttranslationally modified.

Interestingly, the glycoform (*O*-GlcNAc modified higher band) of camel HSPA6 is highly expressed and stabilized under conditions of high or low temperatures as well as hypoxic conditions. On the other hand, the proposed human HSPA6 *O*-GlcNAc modified band is comparatively less in protein level and more influenced by temperature variation as well as hypoxic stresses. Previous studies showed that increased *O*-GlcNAc promotes stress tolerance in many ways, via (i) stabilizing protein conformation [42]; (ii) preventing the formation of protein aggregates [43]; (iii) regulation of glucose metabolism [44] and finally; (iv) changing the activity or the localization of *O*-GlcNac modified HSPs [36,43,45,46,47].

The second chaperone analyzed in our study, CRYAB, is known to undergo *O*-GlcNAc modification [47,48]. However, our data did not reveal *O*-GlcNAc modified forms of both recombinant camel and human CRYAB proteins that bind to *Helix pomatia* lectin. One potential explanation for this variance with the reported *O*-glycosylation could be the oligomeric nature of the α-crystallin under native conditions [49,50] which may hinder the binding of the *O*-GlcNAc attachment sites on CRYAB to the lectin.

HSPA6 is a member of the HSP70 family that has been closely associated with thermal stress. The role of HSPA6 as a chaperone and stress responsive protein has been previously elucidated [24,31,51]. Although its expression is usually low under basal conditions, HSPA6 has been identified as one of the major upregulated HSPs together with HSPA1A following heat stress [52,53]. In fact, HSPA6-lacking cells exhibited reduced viability when treated with high temperature or the proteasome inhibitor MG132 [24]. Members of the HSP70 family showed strong induction and efficient cytoprotection following heat stress in camel compared to other species including human [4,5]. Recently, stress studies concerning camel HSPs in controlled climatic chambers have revealed significant increase of HSPA6 levels following elevated temperature incubations [54].

Given that *O*-GlcNAc modification of cytosolic proteins could influence the whole cellular function in response to extracellular stimuli [36], we suggest that increased *O*-GlcNAc in camel HSPA6 could be associated with a physiological function that does not occur in human HSPA6. However, this hypothesis needs further investigation. Previous studies have addressed the influence of *O*-GlcNAc levels on the function of many proteins where alteration in protein *O*-GlcNAc led to changes in enzyme activities [55,56,57], protein-protein interactions [58,59], protein turnover [58,60] and DNA binding properties [36,61]. Various stimuli were found to increase *O*-GlcNAc levels inside the cells such as heat shock, hypoxia, oxidative stress, ethanol, UV and osmotic stress [36]. 

Our current study provides a strong evidence for differential posttranslational modification of HSPA6 in camel and human upon exposure to hypoxic and thermal stresses. Increased *O*-GlcNAc levels of HSPs in camel are suggested to enhance the functionality of camel HSPs towards extreme cellular stresses.

## 4. Materials and Methods

### 4.1. Immunological Reagents

Anti-c-Myc tag mouse monoclonal antibody (Myc.A7) and horseradish peroxidase-conjugated secondary antibodies were from Thermo Fisher Scientific GmbH (Schwerte, Germany). Anti-β-actin (C4) was purchased from Santa Cruz Biotechnology, Inc. (Heidelberg, Germany). Rabbit anti-calnexin antibody was obtained from Sigma-Aldrich Chemie GmbH (Munich, Germany). Secondary antibodies coupled to Alexa Fluor dyes were obtained from Invitrogen Corporation (Karlsruhe, Germany). 

### 4.2. Sample Collection, RNA Isolation, cDNA Synthesis and Subcloning

Freshly obtained camel (*Camelus dromedaries*) liver tissue from authorized local abattoir in Egypt and human Caco-2 cells were used to isolate total RNA using Trizol reagent (Invitrogen Corporation) according to the manufacturer’s protocol. The extracted RNA was used for cDNA synthesis using RevertAid first strand cDNA synthesis kit (Thermo Fisher Scientific GmbH, Germany). Camel and human cDNA of αB-crystallin and HSPA6 were amplified by PCR using the specific primers as shown in Table 2. Oligonucleotides were provided by Sigma-Aldrich Chemie GmbH (Munich, Germany). The PCR products were cloned into pCR-Zero Blunt vector (Invitrogen Corporation) as an intermediate vector and finally subcloned into the mammalian expression vector pcDNA3.1 Myc His A using specific restriction enzymes. A schematic diagram of the cDNA constructs is shown in Figure 1A.

### 4.3. Transient Transfection of COS-1 Cells, Western Blotting, Biosynthetic Labeling, and Immunoprecipitation

COS-1 cells were cultured in humidified atmosphere containing 5% CO2 at 37 °C in Dulbecco’s Modified Eagle’s Medium (DMEM) containing (10%, *v*/*v*) (fetal calf serum) FCS and 100 U/mL penicillin and 0.1 mg/mL streptomycin. Cells were transfected with plasmid constructs encoding recombinant camel/human HSPA6 or αB-crystallin by the diethylaminoethyl (DEAE)-dextran method [62]. After 2 days, transfected and control non-transfected COS-1 cells were solubilized in 25 mM Tris pH 8 containing 0.1% Triton X-100, 0.5% sodium deoxycholate and 50 mM NaCl supplemented with a combination of protease inhibitors for 1 hour at 4 °C with gentle rotation. Postnuclear lysates were divided into two parts; the first part included equal amounts of the postnuclear lysate that were resolved by SDS-PAGE on 9% slab gels according to Laemmli (1970) [63]. The second major part of the lysate was allowed to bind anti-Myc antibody for 2 h followed by the addition of protein A Sepharose (PAS) 1.5 h at 4 °C. The immunoprecipitates were washed three times as described before [64]. Either lysates or immunoprecipitates were transferred onto polyvinylidene difluoride (PVDF) membrane using wet-blot method. The membrane was blocked with 5% low fat milk solution in phosphate buffered saline (PBS) containing 0.1% Tween 20. After protein transfer to the PVDF membrane, immune detection of camel and human HSPs was performed by anti-Myc primary antibodies followed by HRP-conjugated secondary antibody incubation and washing steps with PBS containing 0.1% Tween 20 after each incubation. Following addition of chemiluminescent substrate, the corresponding bands were detected by a ChemiDoc XRS System (Bio-Rad Laboratories GmbH, Munich, Germany). For biosynthetic labeling, transfected COS-1 cells were labeled using ^35^S-methionine for 4 h and the cells were rinsed, solubilized and the postnuclear lysate was recovered according to the method described by Naim et al. (1991) [62]. Recombinant HSPs were immunoprecipitated from the lysate with anti-Myc-tag antibody and resolved by SDS-PAGE. The gel was then dried on filter paper and the protein signals were visualized on X-ray films as before [62].

### 4.4. Indirect Immunofluorescence Analysis

COS-1 cells were seeded on coverslips and transfected with HSP constructs. After 2 days, the cells were fixed with 4% paraformaldehyde and permeabilized in a blocking solution containing 0.5% Saponin, 0.1% Triton X-100 and 1% (bovine serum albumin) BSA in PBS. The cells were subsequently incubated with primary and then secondary antibodies in blocking solution with washing steps in between. Finally the samples were washed with PBS, dipped in dH_2_O and mounted with ProLong Gold Antifade Reagent containing DAPI to visualize the cell nucleus. Leica TCS SP5 confocal microscope (Leica Microsystems, Wetzlar, Germany) with a HCPL APO 63 × 1.3 oil immersion objective was used to examine samples.

### 4.5. Characterization of the Protein Glycosylation in Camel and Human HSPA6

The postnuclear lysates of COS-1 cells expressing recombinant camel or human HSPA6 were mixed with *Helix pomatia* lectin Sepharose (6 MB, Pharmacia fine chemicals) for 2–3 h with gentle shaking. Thereafter, the lectin beads were washed 2 times with PBS containing 1% Triton-X 100 and the bound proteins were eluted overnight in 10 mM *N*-Acetyl glucosamine at 4 °C with gentle shaking. Recombinant HSPA6 or recombinant CRYAB proteins were isolated from similar cell lysates by immunoprecipitation and used as controls. The samples were finally analyzed by Western blotting using anti-Myc antibody as the primary antibody.

### 4.6. Treatment with Different Temperatures and Low Oxygen Conditions

For temperature treatment, COS-1 cells transiently expressing recombinant camel or human HSPA6 proteins were exposed to 39.5 °C, 37 °C or 20 °C. The incubations were performed in humidified atmosphere without CO_2_ control and in the presence of 20 mM final concentration of HEPES buffer pH 7.4 to avoid pH variations in the medium during treatment. Oxygen control glove box (Coy Laboratory Products, Grass Lake, MI, USA) was used to expose cells to hypoxia at 37 °C, 1% O_2_ and 5% CO_2_ in a humidified (100%) atmosphere. Either one of these treatments were performed for 15 and 24 h post-transfection. Thereafter the cells were lysed and the camel and human HSPA6 were analyzed in the postnuclear lysates by Western blotting.

### 4.7. Statistical Analysis

Immunoblot bands were quantified by the Quantity One 1-D Analysis Software (Bio-Rad Laboratories GmbH) and data analysis was carried out by Microsoft Excel using Prism 5 software. The designated error bars were calculated according to the standard error of the mean (SEM) format from at least 5 independent experiments unless otherwise indicated. Statistical analysis was performed using paired Student *t*-test compared to control. * *p* ≤ 0.05.

## Figures and Tables

**Table 1:** Predicted *O*-GlcNAc attachment sites in camel and human HSPs.

Residue Number	Residue	*O*-Glc-NAc Result ^1^	Potential	Threshold 1	Threshold 2
**Camel CRYAB**					
41	T	+++	0.6512	0.3982	0.4871
42	T	+	0.4726	0.3966	0.4850
43	S	++	0.5398	0.4026	0.4931
53	S	+	0.4490	0.3919	0.4786
134	T	++	0.6134	0.4376	0.5402
135	S	++	0.5522	0.4257	0.5242
153	S	+	0.3793	0.3276	0.3919
170	T	+++	0.6383	0.3557	0.4299
**Human CRYAB**					
41	S	+++	0.6443	0.3921	0.4790
42	T	+	0.4260	0.3923	0.4792
43	S	++	0.4972	0.3990	0.4882
53	S	+	0.4520	0.3907	0.4770
132	T	++	0.5897	0.4157	0.5107
134	T	++	0.5798	0.4332	0.5343
135	S	++	0.5551	0.4190	0.5152
153	S	+++	0.5567	0.3481	0.4196
170	T	+++	0.6383	0.3557	0.4299
**Camel HSPA6**					
40	T	+	0.5184	0.4261	0.5247
160	T	+	0.4538	0.3931	0.4803
275	T	+	0.442	0.4185	0.5145
427	T	+	0.4854	0.424	0.5219
432	T	+	0.5234	0.4549	0.5636
434	S	+	0.4552	0.4344	0.5359
621	S	++	0.5823	0.4446	0.5497
634	S	+	0.4399	0.41	0.5031
635	T	+	0.4453	0.4263	0.525
**Human HSPA6**					
40	T	+	0.5184	0.4261	0.5247
160	T	+	0.4538	0.3931	0.4803
275	T	+	0.442	0.4185	0.5145
427	T	+	0.4854	0.424	0.5219
432	T	+	0.5056	0.4518	0.5594

^1^ *O*-GlcNAc Result: This can be one of five possibilities; No *O*-GlcNAc predicted; *O*-GlcNAc predicted: *(different strengths)*; + Potential > Thresh-1; ++ Potential > Thresh-2 (Thresh-2 is a threshold based on more stringent surface measures); +++ Potential > (Thresh-2 + 0.1); ++++ Potential > (Thresh-2 + 0.1) and Potential ≥ 0.75.

**Table 2:** Primers designed for specific amplification of the cDNAs of target camel and human HSPs.

**Target cDNA**	**(Protein_ID) GenBank**	**Camel HSPs Primers**
CRYAB	AHL21625.1	Forward 5′-ATGGACATCGCCATCCACC-3′ Reverse 5′-CTATTTCTTGGGGGCTGCGG-3′
HSPA6	ADO12067.1	Forward 5′-ATGTCTGCCGCAAAGGAAGTGG-3′ Reverse 5′-TTAATCAACCTCCTCAATAACAGGGCCA-3′
		**Human HSPs Primers**
CRYAB	AAP35416.1	Forward 5′-ATGGACATCGCCATCCACC-3′ Reverse 5′-CTATTTCTTGGGGGCTGCGG-3′
HSPA6	NP_002146.2	Forward 5′-ATGCAGGCCCCACGGGAG-3′ Reverse 5′-TCAATCAACCTCCTCAATGATGGGGCC-3′

**Figure A3:**
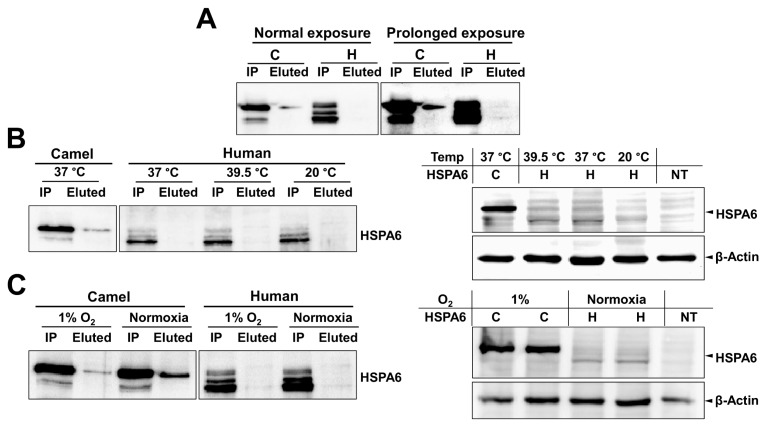
(**A**) cHSPA6 and hHSPA6 transiently expressed in COS-1 cells were subjected to *Helix pomatia* Sepharose as described previously. The lectin-bound material was immunoprecipitated with anti-Myc and analyzed by Western blotting. hHSPA6 did not bind to the lectin in contrast to cHSPA6 (left panel). The right panel shows an overexposed version of the same blot that also shows that no binding of hHSPA6 to the lectin has occurred; (**B**) The lectin pull down assay was repeated under different temperatures as indicated (left panel). No *O*-GlcNAc modified forms of hHSPA6 were detected in contrast to cHSPA6 that bound to the lectin. The right panel shows Western blot of the corresponding total lysates as control; (**C**) Similar to (**B**), the same lectin experiment was performed after different oxygen treatments. Here again no *O*-GlcNAc forms of hHSPA6 in contrast to cHSPA6 that revealed *O*-GlcNAc forms under both hypoxic and normoxic conditions. The Western blot of the corresponding lysates is shown at the right panel as control.

## Abbreviations

HSP	Heat shock protein
*O*-GlcNAc	*O*-linked β-*N*-acetylglucosamine
PTM	Post-translational modification

## Appendix B. The Expression and Mass Spectrometry Analysis of Camel and Human HSPA6

cHSPA6 and hHSPA6 constructs were transfected to COS-1 cells. The cell lysate were extracted as explained previously in the material and methods section followed by IP using Myc antibody as described previously. The immunoprecipitates were then resolved on 9% SDS and stained using colloidal Coomassie stain as described before [65]. The results of the analyzed protein bands are summarized in Table A1 and Figure A2.

**Table A1:** Mass spectrometry results of the analyzed protein bands.

Protein Band	Protein	Score	Protein Mass (kDa)	Unique Peptide Number	Coverage %
a	cHSPA6	10,069	75.8	30	86.44
b	cHSPA6	2416.03	75.8	19	65.31
c	hHSPA6	2112.6	76.2	19	66.62
d	hHSPA6	4407	76.2	23	71.87
e	hHSPA6	6215.7	76.2	29	73.62
